# Identification of a DNA Methylome Profile of Esophageal Squamous Cell Carcinoma and Potential Plasma Epigenetic Biomarkers for Early Diagnosis

**DOI:** 10.1371/journal.pone.0103162

**Published:** 2014-07-22

**Authors:** Xufeng Li, Fuyou Zhou, Chunyu Jiang, Yinuo Wang, Yanqiang Lu, Fei Yang, Nengchao Wang, Haijun Yang, Yanfang Zheng, Jiren Zhang

**Affiliations:** 1 Oncology Center, ZhuJiang Hospital of Southern Medical University, Guangzhou, China; 2 Anyang Cancer Hospital, Anyang, China; 3 Institute of Targeted Molecular Medicine, Shanghai, China; Institut national de la santé et de la recherche médicale, France

## Abstract

DNA methylation is a critical epigenetic mechanism involved in key cellular processes. Its deregulation has been linked to many human cancers including esophageal squamous cell carcinoma (ESCC). This study was designed to explore the whole methylation status of ESCC and to identify potential plasma biomarkers for early diagnosis. We used Infinium Methylation 450k array to analyze ESCC tissues (n = 4), paired normal surrounding tissues (n = 4) and normal mucosa from healthy individuals (n = 4), and combined these with gene expression data from the GEO database. One hundred and sixty eight genes had differentially methylated CpG sites in their promoter region and a gene expression pattern inverse to the direction of change in DNA methylation. These genes were involved in several cancer-related pathways. Three genes were validated in additional 42 ESCC tissues and paired normal surrounding tissues. The methylation frequency of *EPB41L3*, *GPX3*, and *COL14A1* were higher in tumor tissues than in normal surrounding tissues (P<0.017). The higher methylation frequency of *EPB41l3* was correlated with large tumor size (P = 0.044) and advanced pT tumor stage (P = 0.001). The higher methylation frequency of *GPX3* and *COL14A1* were correlated with advanced pN tumor stage (P = 0.001 and P<0.001). The methylation of *EPB41L3*, *GPX3*, and *COL14A1* genes were only found in ESCC patients' plasma, but not in normal individuals upon testing 42 ESCC patients and 50 healthy individuals. Diagnostic sensitivity was increased when methylation of any of the 3 genes were counted (64.3% sensitivity and 100% specificity). These differentially methylated genes in plasma may be used as biomarkers for early diagnosis of ESCC.

## Introduction

Esophageal cancer (EC) is the eighth most common cancer worldwide and the sixth most common cause of death from cancer [1]. Esophageal cancer usually occurs as either squamous cell carcinoma in the middle or upper one-third of the esophagus, or as adenocarcinoma in the lower one-third or junction of the esophagus and stomach. In the highest risk area, which stretches from northern Iran through the central Asian republics to North-Central China and often referred to as the “esophageal cancer belt”, 90% of cases are squamous cell carcinomas [2]. Esophageal squamous cell carcinoma (ESCC), which is the major histological type of esophageal cancer, is one of the most aggressive malignant tumors. Despite advances in diagnostic methods and combined treatment modalities, the majority of esophageal squamous cell carcinomas (ESCC) are diagnosed at advanced stages and overall 5-year survival rate is still poor. In sharp contrast, the 5-year survival rate for early-stage ESCC patients was 100% after endoscopic mucosectomy. Therefore, it is imperative to further understand the underlying molecular mechanism of ESCC, and to identify effective biomarkers for early diagnosis and potential targets for prevention and therapy.

Epigenetics, one of the most promising and expanding fields in current biomedical research, refers to stable alterations in gene expression with no underlying modifications in the genetic sequence. Both genetic and epigenetic aberrations are linked by intricate crosstalk, and can either individually or in synergy lead to the development of cancer. One study shows that up-regulation of FOXM1 in normal human cells can orchestrate a DNA methylation signature that mimics the cancer methylome landscape [3]. Accumulating evidence suggests that epigenetic changes such as alterations in DNA methylation play a crucial role in ESCC [4], [5]. Numerous tumor-related genes, including *FHIT* [6], *RARβ* [6]–[8], *CDKN2A* [7], [9], [10], *MGMT* [7], [9], [11], *RASSF1* [6], *RBP1* [7], [8], *CDKN2AB* [10], *MT1G* [7], *MLH1* [10], [12], *CDH1* [13], *VHL* [6], *MT3* [14], *C2orf40* [15], *RPRM* [16], *CLND3* [7], *APC* [17], and *UCHL1* [18], which are involved in various carcinogenic pathways, have been revealed to be frequently hypermethylated in ESCC. However, these studies [6]–[18] are focused on a limited number of genes and did not interrogate genome wide changes in DNA methylation. Therefore, a large-scale analysis of DNA methylation profiles during esophageal carcinogenesis could provide not only a better understanding of the molecular pathways involved in this process, but could also facilitate the identification of potential targets for biomarker discovery and therapeutic intervention. Lima etc. did the first study to address methylation changes in ESCC in a large set of genes, but the microarray they used is the Illumina GoldenGate Methylation assay which only covers 1505 CpG sites [19]. Infinium Methylation 450K array (Illumina, San Diego, CA, USA) is a newly developed BeadChip platform, which can test more than 480,000 individual CpG sites in the human genome. The high correlation between Infinium Methylation 450K array data and whole-genome bisulfate sequencing data indicates that this new BeadChip can provide reliable DNA methylation data for epigenomic profiling studies [20].

Furthermore, aberrant DNA methylation usually occurs somatically in cancers and can be detected in the blood circulation, so it may serve as a novel marker for cancer. For example, the methylation of some tumor related genes such as p16, DAPK, RAR-β, CDH1, and RASSF1A have been detected in circulating cell-free DNA [21].

In the current study, we analyzed global methylation profiling of ESCC and normal adjacent tissue in Chinese cancer patients and esophageal mucosa from Chinese healthy individuals, using Infinium Methylation 450K array. After analysis of the methylation differences and then in combination with independent gene expression data using BRB (Biometrics Research Branch)-Array Tools of the National Cancer Institute, a set of genes that are deregulated by aberrant DNA methylation in ESCC was identified. We then focused on 3 aberrant DNA methylation genes—*EPB41L3*, *GPX3*, and *COL14A1*, with validation analysis using additional ESCC tumor tissues and adjacent normal tissues. In order to evaluate whether the methylation status of the 3 candidate genes is useful for diagnosing ESCC, we also tested the methylation of the circulating cell-free DNA in patients' plasma and controls.

## Patients and methods

### Clinical samples

All ESCC patients and normal volunteers were gathered from Anyang Cancer Hospital, Henan province, China, from June to September in the year 2012. Tumors and paired adjacent non-tumor tissue samples from four ESCC patients were used for Infinium Methylation 450k array analysis. These 4 patients are relatively early stage patients (stage IA to stage IIA), and their clinical characters are listed in Table 1. Following surgery, the tissue samples were promptly frozen in liquid nitrogen and then stored at −80°C until use. Additional 42 ESCC patients were gathered to acquire blood samples before surgery, tumor and adjacent non-tumor tissues following surgery. These patients included 27 males and 15 females whose ages ranged from 42 to 74 years (mean ± SD, 63±7.36), with 8 IIIA, 12 IIB, 16 IIA, and 6 I B patients. None of the patients had a history of chemotherapy or radiotherapy before surgery. The diagnosis was confirmed as squamous cell carcinoma by pathological examination. Tumor stages were evaluated according to the TNM classification of the American Joint Committee on Cancer (AJCC). Four tissue samples collected from healthy volunteers who have no upper digestive tract cancer diagnosed by endoscope, were used for Infinium Methylation 450k array analysis.

**Table 1:** Clinic information of the 4 ESCC patients whose samples were used for methylation array.

specimen	Age (year)	gender	TNM*	G(histologic grade)	Tumor location	stage
T1 and S1	61	male	T2N0M0	G1	Lower	I B
T2 and S2	63	male	T1N0M0	G2	Middle	I B
T3 and S3	65	female	T1N0M0	G1	Middle	I A
T4 and S4	65	male	T2N0M0	G1	Middle	II A

*Tumor stages were evaluated according to the TNM classification of the American Joint Committee on Cancer (AJCC).

Blood samples were collected from 42 patients before surgery as metioned above, and plasma was separated twice by centrifugation at 1000× g for 5 min and stored at −80°C until use. In addition, plasma samples from 50 healthy volunteers were used as negative controls. The healthy volunteers (24 males and 26 females) ranged in age from 17 to 75 years (mean ± SD, 52.7±11.5). All participants signed an informed consent and information was obtained using a standardized questionnaire, including data on tobacco smoking, alcohol drinking, and family history. An individual was classified as a smoker if he or she smoked at least one cigarette per day for more than one year. An individual was classified as a drinker if he or she drank alcoholic beverages five times per week for more than one year. Family history of cancer was defined as positive when at least one of the patient's first-degree relatives was definitely diagnosed with cancer.

This study was approved by the ethics committee of the Anyang Cancer Hospital in Henan province in China. Written Informed consent have been obtained from all the participants and kept as records submitted back to ethics committee. For minor participants less than 18 years old (2 healthy volunteers aged 17), written informed consent was obtained from guardians. The ethics committee approved the consent procedure.

### DNA methylation assay

Genomic DNA was extracted from tissue samples using the QIAmp DNA Mini Kit (Qiagen, Hilden, Germany). Cell-free plasma DNA was isolated using QIAmp DNA Blood Mini Kit (Qiagen, Hilden, Germany). Extracted DNA was bisulfite-modified using EZ-DNA Methylation-Gold Kit (Zymo Research, Orange, CA, USA).

Genome-wide DNA methylation profiling was performed using the Infinium Methylation 450K array (Illumina, San Diego, CA, USA). Genomic DNAs were modified with the EZ-DNA methylation kit (Zymo Research, Orange, CA, USA) following recommendations from Illumina. The Illumina Infinium assay was also conducted according to the manufacturer's protocol.

Methylation-specific PCR (MSP) was used to examine the methylation status of *EPB41L3*, *GPX3*, and *COL14A1*, as described previously in the literature [22]. The primer sequences of *EBP41L3* were described in the literature [23]. The primer sequences of *GPX3* and *COL14A1* were designed by an online tool as described in the literature [24]. Primer sequences and conditions are listed in Table 2. SssI-treated normal lymphocyte DNA was used as positive control. Positive controls and negative controls (without DNA) were performed in each set of MSP and each MSP was repeated three times. The MSP products were separated electrophoretically on 2% agarose gels.

**Table 2:** Primer and Probe Sequences of Genes Related to Esophageal Squamous Cell Carcinoma.

Genes		Primer sequences	Annealing temperature (°C)	Product size (base pair)
**EPB41L3**	M	Sense:TTGGTTTTTTTCGTACGGTT	62	110
		Antisense:AACCCAAAACTACTCGCCGCT		
	U	Sense:AGGTTGGTTTTTTTTGTATGGTT	64	114
		Antisense:AACCCAAAACTACTCACCACT		
**GPX3**	M	Sense:CGTTCGTTTTTGAAATTTTAGTC	62	140
		Antisense:CTACCTAATCCCTAACCACCGT		
	U	Sense:TGTTTGTTTTTGAAATTTTAGTTGT	62	140
		Antisense:CTACCTAATCCCTAACCACCATC		
**COL14A1**	M	Sense:GTGAATGGGTGTTTTTTTAGATTTC	62	167
		Antisense:AACGCCTTTCGACTTCTACG		
	U	Sense:GAATGGGTGTTTTTTTAGATTTTGT	62	168
		Antisense:CAAAACACCTTTCAACTTCTAGACT		

### Expression data of ESCC

Gene expression data (GSE20347) of ESCC that was based on the Affymetrix array were downloaded from Gene Expression Omnibus of National Center for Biotechnology Information (NCBI) (http://www.ncbi.nlm.nih.gov/geo/query/acc.cgi?acc=GSE20347), a public data repository [25]. These expression data generated using 17 tumor tissues and 17 paired adjacent normal tissue. The downloaded data was imported to BRB-Array Tools for statistical analysis.

### Statistical analysis

Raw Infinium Methylation 450K array data, which have been deposited in NCBI's Gene Omnibus (GEO) repository and are accessible through GEO number GSE52826, were obtained using GenomeStudio software (Illumina) after scanning the BeadChips. Differentially methylated CpG sites were identified by analyzing the CpG island microarray data with the class comparison feature of BRB-Array Tools (http://linus.nci.nih.gov/BRB-ArrayTools.html). Unsupervised hierarchical clustering analysis was also conducted using the BRB-Array tool. M-value, which is the log_2_ ratio of methylated probe intensity and unmethylated probe intensity, is used to measure the methylation level. M-value method is approximately homoscedastic in the entire methylation range, so it is more statistically valid in differential and other statistic analysis [26]. In addition, we also analyzed the expression data of ESCC (GSE20347) with the class comparison tool of BRB-Array tools.

Association between each phenotype and DNA methylation at each CpG site was tested separately within the Infinium Methylation 450K array data. We determined whether each individual CpG site was statistically significant based on the false discovery rate (FDR) in order to correct possible false positives from multiple tests (α = 0.05). We also subsequently calculated a fold change of M value that had greater or equal to 2 between 2 comparison groups. Therefore, a differentially methylated CpG (DMC) was defined as a CpG site with a FDR-adjusted P value <0.05 and a fold change of M-value between 2 comparison groups greater or equal to 2. Similarly, we defined the difference of expression data as a FDR adjusted P value <0.05 and a fold change between 2 comparison groups greater or equal to 2. Pathway analysis was performed using DAVID Bioinformatics Resource.

IBM SPSS Statistics 20.0 (IBM Corp., Armonk, NY, USA) was used for other statistical analysis. Statistical analysis was performed using Pearson chi-square test or the Fisher exact test for differences between groups. ROC curve analysis was used to determine AUC, sensitivity, and specificity of circulating cell-free plasma DNA methylation. P value <0.05 were considered statistically significant. All reported P values were based on 2-sided tests. Bonferroni adjustments were estimated to correct for multiple tests.

## Results

### Identification of differentially methylated CpG sites in ESCC

**Figure 1:**
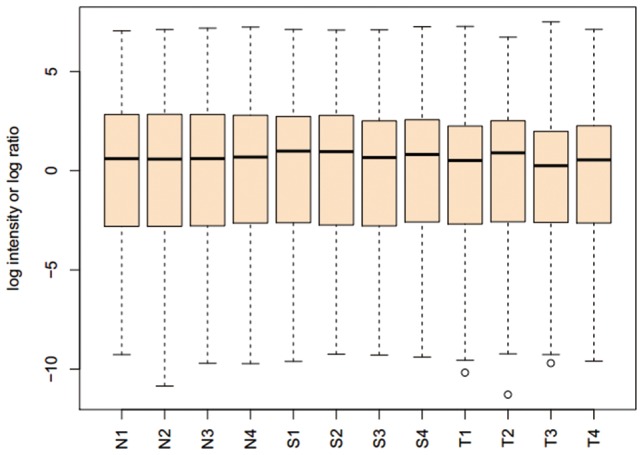
Boxplots of log ratio of 12 samples' raw microarray data. After the log_2_ transfer and normalization, all samples showed very similar signal distributions.

**Figure 2:**
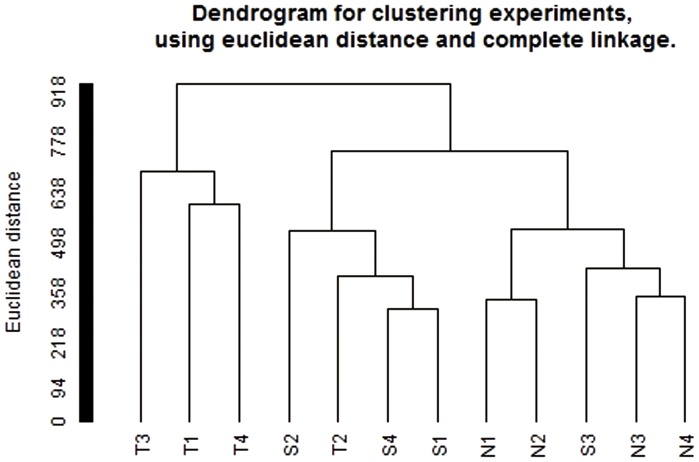
Unsupervised clustering of samples. Unsupervised hierarchical clustering on the entire DNA methylation data set showed the 2 clusters which were mainly composed by two different phenotypes: tumor and normal (including normal and adjcent normal tissues). This indicated that ESCC and normal samples might have different DNA methylation characters and patterns. In addition, there were some differences in DNA methylation profiles between adjacent normal and normal tissues.

Genome-wide DNA methylation profiling was conducted using the Infinium Methylation 450K array. We evaluated 4 paired tumor samples and corresponding adjacent normal tissues, along with 4 normal tissue samples from healthy volunteers that were originally collected from Anyang Cancer Hospital in Henan province in China. The methylation array data was submitted to Gene Expression Omnibus (GEO), NCBI (National Center for Biotechnology Information) (data number GSE52826). After normalization, all 12 samples showed very similar signal distributions (Figure 1), which means with both hypermethylation and hypomethylation changes, tumor, adjacent normal and normal tissues are similar on overall methylation signals. While unsupervised hierarchical clustering on the entire DNA methylation data set showed the 2 clusters which were mainly composed by two different phenotypes: tumor and normal (including normal and adjcent normal tissues) (Figure 2). This indicated that ESCC and normal samples might have different DNA methylation characters and patterns. In addition, the results also showed that there were some differences in DNA methylation profiles between adjacent normal and normal tissues (Figure 2).

**Figure 3:**
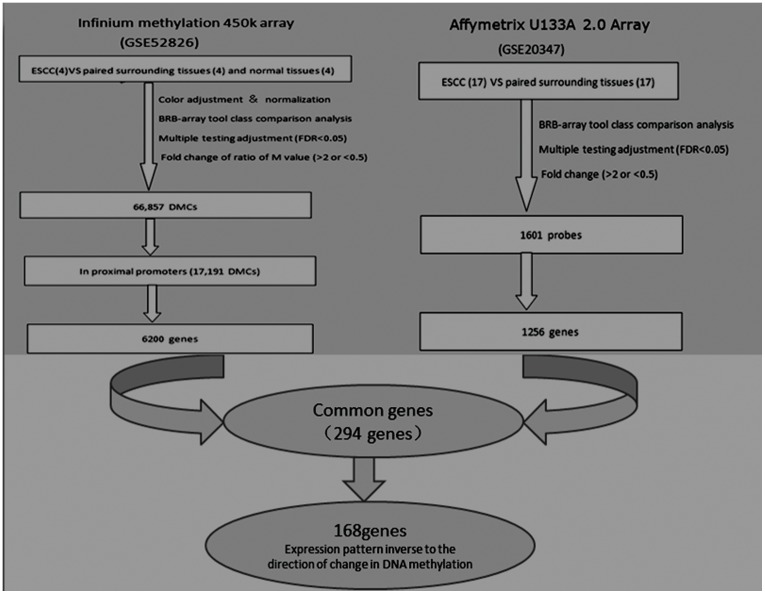
Combination analysis between DNA methylation data and gene expression data identified 168 differentially methylated genes in ESCC. Methylation data analysis showed 66,857 DMCs, which related to 6,200 genes (gene set 1). Expression data analysis showed 1,601 probe differences between ESCC and control, which related to 1.256 genes (gene set 2). There were 294 common genes between gene set 1 and gene set 2. 168 genes were selected due to inverse correlation between DNA methylation and expression change.

To identify aberrant differential methylated CpG (DMC) in ESCC, we performed statistical analysis to pare down the selection of CpG sites based on DNA methylation differences (Figure 3). We put normal and adjacent normal (non-tumor) tissues into one group and compared with tumor tissues. A total of 66,857 CpG sites showed statistical significance with false discovery rate (FDR) adjusted P<0.05 and at least two-fold methylation change between ESCC and normal/adjacent normal samples. These 66,857 DMCs included 18,146 hypermethylated CpG sites (hyper-DMCs) and 48,711 hypomethylated CpG sites (hypo-DMCs) in tumors compared with normal/adjacent normal tissues. Interestingly, 75.3% of hyper-DMCs (13,656 CpG sites) were located in CGI, CGI shores, or CGI shelves, but only 37.3% of hypo-DMCs were found in these regions.

### Combination analysis between methylation data and expression data of ESCC

**Figure 4:**
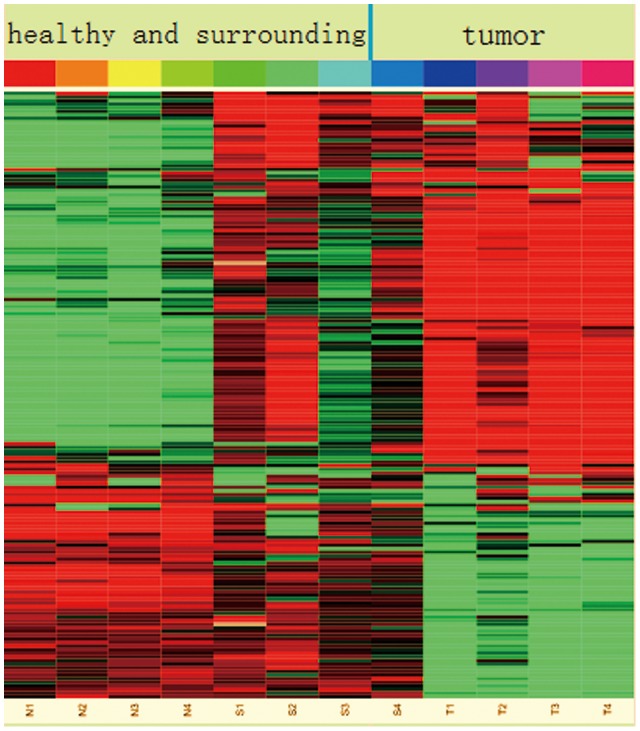
Unsupervised gene cluster of 168 genes. The heat map of these genes shows different methylation between tumor tissues and normal tissues. Red indicates hypermethylation, while green indicates hypomethylation.

To find aberrant DNA methylation that may cause gene expression change in ESCC, we correlatively analyzed methylation data and expression data of ESCC downloaded from the GEO database. The methylation data analysis showed that a total of 66,857 DMCs included 17,197 CpG sites in proximal promotes, which were related to 6,200 genes (gene set 1). The expression data compared 17 ESCC tumor tissues and 17 paired adjacent normal tissues, and showed that there were 1,601 probe difference between ESCC and control, which were related to 1256 genes (gene set 2). There were 294 common genes between gene set 1 and gene set 2. Among the 294 genes, 168 genes showed inverse correlation between DNA methylation and expression change, which means when compared with normal tissue, they were either hypermethylated with lower expression, or hypomethylated with higher expression in tumor tissue (Figure 3). The heat map figure showed that these 168 genes were differently methylated between tumor and normal tissues (Figure 4). In addition, Figure 4 also showed that there were some differences in DNA methylation profiles between adjacent normal and normal tissues.

Based on the combination analysis result of the 168 genes, we further identified the pathway of these genes. The DAVID (Database for Annotation, Visualization, and Integrated Discovery v6.7) online bioinformatics analysis showed that these genes were majorly involved in 6 pathways in the KEGG (Kyoto Encyclopedia of Genes and Genomes) database, which include “focal adhesion”, “ECM-receptor interaction”, “axon guidance”, “pathways in cancer”, “apoptosis”, and “aldosterone-regulated sodium reabsorption”.

### Promoter hypermethylation profile of *EPB41L3*, *GPX3*, and *COL14A1* in ESCC and correlation with clinico-pathological parameters

**Figure 5:**
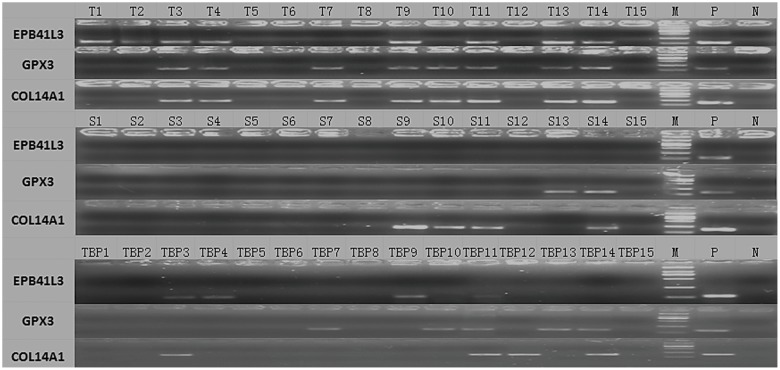
Representative results of MSP analysis of the candidate genes. M, markers; N, negative control (water blank); P, positive control (universal methylated DNA); T, tumor tissue; S, paired surrounding normal tissue; TBP, blood plasma of tumor patients. Samples were scored as methylated when there was a clearly visible band with the methylated primers.

As we described above, the 168 genes were either hypermethylated with lower expression, or hypomethylated with higher expression in tumor tissue compared with normal tissue. We then calculated the ESCC to control ratio of DNA methylation and gene expression rate for each gene (data not shown), and picked up 3 genes with most significant difference between tumor and normal. *EPB41L3*, *GPX3*, and *COL14A1* were chosen. We used methylation specific PCR (MSP) analysis to study DNA methylation status of *EPB41L3*, *GPX3*, and *COL14A1* genes in tumor and paired surrounding normal tissue from 42 ESCC patients (Figure 5). The unmethylated form of all genes was detected in all samples. The MSP analysis revealed that the methylation frequencies of *EPB41L3*, *GPX3*, and *COL14A1* in ESCC tumor samples were 59.5%, 54.8%, and 45.2%, respectively. The methylation frequencies of *EPB41L3*, *GPX3*, and *COL14A1* in normal surrounding tissue were 4.8%, 9.5%, and 11.9%, respectively. The frequencies of hypermethylated genes were significantly higher than that in paired surrounding normal tissues (P<0.01) (Table 3).

**Table 3:** Frequency of Promoter Methylation for Individual Genes in Esophageal Squamous Cell Carcinoma Patients (n = 84).

Genes	Frequency of Methylation(%)		P value
	ESCC†	ST‡	
***EPB41L3***	59.5	4.8	<0.001*
***GPX3***	54.8	9.5	<0.001*
***COL14A1***	45.2	11.9	0.001*

† ESCC, esophageal squamous cell carcinoma;

‡ ST, surrounding normal tissue;

*statistical significance after multiple testing (P<0.01).

The relationship between the clinico-pathological parameters and DNA methylation patterns in the testing group cases was explored. Methylation of *EPB41L3* was more frequently seen in patients with tumor size 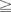
5 cm than in patients with tumor size <5 cm (P = 0.044, Table 4). We also found that methylation of *EPB41L3* correlated significantly with the advanced pathologic tumor (pT) classification (P = 0.001, Table 4). The methylation frequencies of *GPX3* were lower in the pT1-2 and pN0-1 groups compared with the pT3 and pN2 groups, respectively. For the methylation of *COL14A1*, we observed a significant difference between males and females (P = 0.019, Table 4). In addition, methylation of *COL14A1* was much more frequent in the pN2 group compared to the pN0-1 group (P = 0.000, Table 4). We also found that the methylation of *COL14A1* was higher in moderately differentiated tumors than in well and poorly differentiated tumors. No significant correlations were observed between methylation of any gene with age, family history, smoking, or drinking.

**Table 4:** Correlation Between DNA Hypermethylation and Clinico-pathological Parameters of Esophageal Squamous Cell Carcinoma Patients (n = 42).

	Frequency of methylation (%)		
	EPB41L3	GPX3	COL14A1
**Gender**			
Male(n = 27)	55.6	51.9	44.4
Female(n = 15)	66.7	60.0	46.7
P value	0.482	0.611	0.890
**Age group(year)**			
<60 (n = 11)	72.8	63.6	36.4
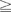 60 (n = 31)	54.8	51.6	48.4
P value	0.299	0.726	0.726
**Family history**			
Yes(n = 17)	60.0	56.0	48.0
No(n = 25)	58.8	52.9	41.2
P value	0.939	0.845	0.663
**Tumor size**			
<5(n = 27)	48.1	48.1	44.4
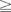 5(n = 15)	80.0	66.7	46.7
P value	**0.044**	0.248	0.890
**Differentiation**			
Well (n = 6)	50.0	16.7	16.7
Moderately (n = 29)	62.1	65.5	58.6
Poorly (n = 7)	57.1	42.9	14.3
P value	0.854	0.064	**0.025**
**pT category**			
T1–T2 (n = 15)	26.7	26.7	26.7
T3 (n = 27)	77.8	70.4	55.6
P value	**0.001**	**0.006**	0.071
**pN category**			
N0–N1(n = 34)	52.9	44.1	32.4
N2(n = 8)	87.5	100	100
P value	0.056	**0.001**	**0.000**
**Smoking** *			
Smoker(n = 18)	44.4	55.6	44.4
Non-smoker(n = 23)	62.5	52.2	43.5
P value	0.105	0.829	0.951
**Alcohol** *			
Drinker(n = 9)	44.4	33.3	33.3
Non-drinker(n = 32)	62.5	59.4	46.9
P value	0.450	0.260	0.706

*clinical data was not available for one patient.

### Hypermethylation status of *EPB41L3*, *GPX3*, and *COL14A1* in circulating cell-free plasma DNA and correlation with clinico-pathological parameters

The methylation status of *EPB41L3*, *GPX3*, and *COL14A1* genes were assessed in circulating cell-free plasma DNA in the 42 ESCC patients and 50 healthy individuals. The methylation frequency of *EPB41L3*, *GPX3*, and *COL14A1* were 31.0%, 40.5% and 31.0% in cancer patients' plasma, respectively. Examples of MSP analysis are shown in Figure 5. We did not find methylation of the 3 genes in healthy individuals' plasma. There were statistically significant differences of methylation of the 3 genes in circulating cell-free plasma DNA between cancer patients and healthy individuals group (Table 5).

**Table 5:** Frequency of Promoter Methylation for Individual Genes in Blood Plasma of Esophageal Squamous Cell Carcinoma Patients and Controls.(n = 92).

Genes	Frequency of Methylation (%)		P value
	TBP†	NBP‡	
***EPB41L3***	31.0	0	0.000*
***GPX3***	*40.5*	*0*	*0.000* *
***COL14A1***	31.0	0	0.000*

† TBP, blood plasma of tumor patients;

‡ NBP, blood plasma of normal healthy individuals;

* statistical significance after multiple testing (P<0.01).

We analyzed whether plasma DNA methylation was correlated with clinico-pathological parameters in 42 ESCC patients. Comparing pT3 with the pT1-2 group, we found that the methylation frequencies of *GPX3* and *COL14A1* were significantly higher in the pT3 group (P = 0.044 and P = 0.015, respectively) (Table 6). *EPB41L3* hypermethylation was correlated with advanced pN classification (pN2) (87.5% vs. 17.6%) (P<0.001) (Table 6).

**Table 6:** Correlation between Blood Plasma DNA Hypermethylation and Clinico- pathological Parameters of Esophageal Squamous Cell Carcinoma Patients (n = 42).

	Frequency of methylation (%)		
	EPB41L3	GPX3	COL14A1
**Gender**			
Male (n = 27)	29.6	33.3	25.9
Female (n = 15)	33.3	53.3	40.0
P value	1.000	0.206	0.488
**Age group (year)**			
<60 (n = 11)	27.3	54.5	18.2
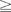 60 (n = 31)	32.3	35.5	35.5
P value	1.000	0.305	0.453
**Family history**			
Yes (n = 17)	32.0	40.0	24.0
No (n = 25)	29.4	41.2	41.2
P value	0.859	0.939	0.237
**Tumor size**			
<5 (n = 27)	29.6	29.6	25.9
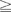 5 (n = 15)	33.3	60.0	40.0
P value	1.000	0.055	0.488
**Differentiation**			
Well (n = 6)	0	16.7	16.7
Moderate (n = 29)	37.9	44.8	37.9
Poor (n = 7)	28.6	42.9	14.3
P value	0.078	0.400	0.312
**pT category**			
T1–T2 (n = 15)	26.7	20.0	6.7
T3 (n = 27)	33.3	51.9	44.4
P value	0.739	**0.044**	**0.015**
**pN category**			
N0–N1 (n = 34)	17.6	35.3	29.4
N2(n = 8)	87.5	62.5	37.5
P value	**0.000**	0.235	0.686
**Smoking** Statis*			
Smoker (n = 18)	27.8	33.3	27.8
Nonsmoker (n = 23)	30.4	47.8	34.8
P value	0.853	0.350	0.632
**Alcohol** Consumption*			
Drinker (n = 9)	11.1	33.3	22.2
Nondrinker (n = 32)	34.4	43.8	34.4
P value	0.240	0.711	0.692

*clinical data was not available for one patient.

### Evaluation of circulating cell-free plasma DNA methylation markers for early diagnosis of ESCC

To assess the clinical usefulness of plasma DNA methylation patterns as early diagnostic biomarkers of ESCC, we calculated their sensitivity and specificity using ROC (receiver operative characteristic) curve analysis. The sensitivity of the methylation status of *EBP41L3*, *GPX3*, and *COL14A1* genes were 31.0%, 40.5%, and 31.0%, respectively. The specificity of any of the 3 genes was 100% based on our data. The overall diagnostic sensitivity of 3 genes for ESCC, when at least 1 of the 3 genes was positive, rose to 64.3%, and the specificity remained 100% (AUC [area under the ROC curve] = 0.821, 95% CI [confidence interval] = 0.727–0.916, Table 7).

**Table 7:** Diagnostic Information of Plasma DNA Methylation of Individual Genes.

Gene	AUC* (95%, CI†)	Sensitivity (%)	Specificity (%)
***EPB41L3***	0.655(0.539–0.770)	31.0	100
***GPX3***	0.702(0.591–0.814)	40.5	100
***COL14A1***	0.655(0.539–0.770)	31.0	100
**Any of the 3 genes**	0.821(0.727–0.916)	64.3	100

*AUC, area under ROC curve;

† CI, confidence interval.

## Discussion

Although epigenetic events have been implicated in esophageal cancer, genome-wide epigenetic deregulation and precise targets of aberrant DNA methylation during the development and progression of ESCC have not been defined. In this pilot study, we describe the methylation profile of Chinese ESCC patients. One hundred and sixty eight differentially methylated genes that were functionally deregulated in ESCC were identified. We validate methylation status of 3 genes from the 168 genes in independent tumor and normal samples and evaluate their value for diagnosis of ESCC.

In this study, we have established a large-scale profile of promoter methylation in Chinese ESCC patients. We combined analysis the Infinium HD 450K methylation array data with gene expression array data. From this we identified a set of genes that have potential functional consequences owing to the aberrant promoter DNA methylation. The pathway analysis showed that these genes take part in tumor-related pathways which may play an important role in the development and progression of the disease. We chose 3 genes from 168 genes for validation in independent tissue samples and evaluated their significance in clinical diagnosis of ESCC. Therefore, our results not only strengthen the findings on the importanance of aberrant DNA methylation in ESCC, but also provide a novel and more comprehensive signature of ESCC methylation.

We tested DNA methylation in more than 480,000 CpG sites using the Infinium HD 450K methylation array in ESCC samples, adjacent normal surrounding tissues, and normal mucosa of healthy individuals. An initial, unsupervised hierarchical analysis with all of the tested CpG sites showed that there are two main clusters: tumor and normal (including adjacent normal surrounding tissues and normal tissues from healthy individuals) (Figure 2). This indicates that tumors show different methylation profiles in comparison with normal tissues. This result supports the previous study of the ESCC DNA methylation profile using the GoldenGate bead array (Illumina, San Diego, CA, USA), which contains 1,505 CpG sites in 807 cancer-related genes [19]. Interestingly, our result also shows that adjacent normal surrounding tissues also have different DNA methylome patterns compared with normal tissues. We presume there may be field cancerization phenomenon existed in adjacent normal tissues. More studies need to be done to explore the detail mechanism of methylation difference between normal and adjacent normal tissues.

BRB-Array Tools is an integrated software system for the comprehensive analysis of DNA microarray experiments [27]. By class comparison using the BRB-Array Tools, we identified 66,857 differentially methylated CpG sites in ESCC. We chose to limit our analysis to the proximal promoter region because this region is well characterized for its effects of DNA methylation on gene silencing. This approach also allowed us to evaluate associations with gene expression changes based on an independent ESCC data set. The assumption is that inverse correlations between promoter methylation and gene expression may plausibly indicate a functional result of differential methylated genes identified in ESCC. This approach identified 168 genes in ESCC. Based on the combined analysis signature of methylation data and expression data, we identified the pathways that are specifically altered in this type of cancer. Our results suggested that these altered pathways may play pivotal role in ESCC tumorigenesis.

A candidate tumor suppressor gene, *EPB41L3*, on 18p11.3 was initially isolated by Tran et al using differential display analysis in a panel of non–small cell lung carcinoma when compared to matched normal tissue [28]. A growing body of evidence supports the role of tumor suppression of the *EPB41L3* gene [29]. *EPB41L3* interacts with cytoplasmic adaptor protein 14-3-3 [30], which is involved in signal transduction, cell cycle regulation, apoptosis, and malignancy [31], and may play a role in growth suppression. The expression of *EPB41L3* was greatly reduced in many kinds of tumors, including non-small cell lung cancer [28], meningiomas [32], breast cancer [33], [34], renal clear cell carcinoma [35], and ovarian cancer [36]. Restoration of *EPB41L3* expression in non-small cell lung cancer or breast cancer cell lines significantly suppressed cell growth *in vitro* [28], [33], and re-expression of *EPB41L3* can induce extensive apoptotic cell death in ovarian cancer cells [36]. Recent studies show that the promoter methylation of *EPB41L3*, leading to loss of its expression, is an important molecular event in several types of tumor cells, whereas *EPB41L3* expression can be restored by a demethylating agent [23], [34], [37]. Nevertheless, the promoter methylation of *EPB41L3* and the function of this gene has not been investigated in ESCC. By combination analysis of the methylation array data and expression data, we found 13 hypermethylated CpG sites in the promoter region of *EPB41L3* and a greater than ten fold change in down-regulation upon comparison between tumor tissues and normal tissues. This result suggests in ESCC the expression of *EBP41L3* is decreased due to its promoter methylation, which is consistent with the previous studies in other types of tumors [23], [34], [37].

Our results show that the promoter methylation frequency of *EPB41L3* in tumor tissues is significantly higher than in the adjacent normal surrounding tissues (59.5% vs 4.8%, P<0.001). Similar results were observed in non-small cell lung cancer and cervical cancer [23], [38]. We also found that the methylation frequency of *EPB41L3* is associated with tumor size and the frequency is higher in larger tumors (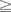
5 cm) than in smaller tumors (<5 cm) (P = 0.044). This result suggests that the methylation of *EPB41L3* may be involved in the proliferation of cancer cells. In addition, our results show that 77.8% of the pT3 samples are methylated, which is significantly higher than 26.7% in pT1-pT2 samples (P = 0.001). This result suggests that the methylation of *EPB41L3* may play an important role in ESCC tumor invasion.

Glutathione peroxidase 3 (GPX3) catalyzes the reduction of peroxides at the expense of glutathione and protects cells against oxidative damage. Thus, the silencing of GPX3 may impair defenses against endogenous and exogenous genotoxic compounds, which could increase gene mutation rates. GPX3 has been found to be frequently hypermethylated in prostate cancer [39], esophageal adenocarcinoma [40], [41], and gastric cancer [42], [43]. Furthermore, the promoter hypermethylation of GPX3 was correlated with the down-regulation of mRNA and/or protein expression. In addition, the mRNA expression can be restored by demethylated agents [41]–[43]. Moreover, suppressive activity of tumor growth and metastasis was demonstrated by both *in vitro* and *in vivo* studies [39]. Our result showed that the methylation frequency in tumor tissues is 54.8%, which is significantly higher than 9.5% in paired adjacent normal surrounding tissues. This result is consistent with a previous study that found that the methylation of GPX3 promoter was more frequent in ESCC tumor tissues (71.4%) than in adjacent nontumor tissues (10.7%) [44]. In addition, we analyzed the correlation of the methylation of GPX3 promoter and clinico-parameter of the patients. We found that the frequency of methylation is higher in pT3 patients and pN2 patients compared with pT1-T2 and pN0-N1 patients. These results suggest that the methylation of GPX3 may be involved in the progression and lymphnoid metastais in ESCC.

*COL14A1* is a large extracellular matrix glycoprotein associated with mature collagen fibrils. Alterations in extracellular matrix composition have been implicated in tumor progression and metastasis. *COL14A1* interacts with decorin [45], a small leucine-rich proteoglycan, which has incrementally been shown to be a powerful inhibitor of growth in a wide variety of tumor cells. This effect is specifically mediated by the interaction of decorin core protein with the epidermal growth factor receptor (EGFR) and other ErbB family proteins [46]. Previous studies have shown that aberrant *COL14A1* DNA methylation has been tested in renal cancer cell lines and primary renal cancers, and the methylation correlated with silencing or down-regulating of mRNA expression [47], [48]. Moreover, RNAi-induced reduced expression of *COL14A1* resulted in the growth of renal cancer cells *in vitro* [48]. In our study, we observed that the methylation frequency of *COL14A1* was significantly higher in tumor tissues than that in adjacent normal surrounding tissues (P = 0.001). We further analyzed the correlation between the methylation status of *COL14A1* and the clinico-parameters. We found that the moderate pathologically differentiated samples have a higher methylation frequency of *COL14A1*, compared with well or poor pathologically differentiated samples. In addition, we also found that the frequency of *COL14A1* was 100% in pN2 patients, which was higher than 32.4% in pN0–N1 patients(P<0.001). This result suggests that aberrant methylation of *COL14A1* may be associated with the lymph node metastasis of ESCC.

Detecting cell-free nucleic acid in plasma or serum could be useful for numerous diagnostic applications and might prevent the need for tumor tissue biopsies. The release of nucleic acids into the blood is thought to be related to the apoptosis and necrosis of cancer cells in the tumor microenvironment. Secretion has also been suggested as a potential source of the nucleic acids [49]. Changes in the levels of circulating nucleic acids have been associated with tumor burden and malignant progression. Several studies have revealed the presence of methylated DNA in the serum or plasma of patients with various types of malignancy, including bladder cancer, breast cancer, cervical cancer, colorectal cancer, hepatocellular carcinoma, lung cancer, non-Hodgkin lymphoma, melanoma, ovarian cancer, pancreatic cancer, and prostate cancer [49]. In our study of the methylation status of *EPB41L3*, *GPX3* and *COL14A1* in the plasma of ESCC patients and healthy individuals, the methylation frequencies of these genes are all greater than 30% in the plasma of the ESCC patients. However, absolutely no methylation was found in the plasma of healthy individuals, which may be due to several reasons and not a true case. One limitation of our study is that we used MSP as test method here, which is not quatitative and not sensitive enough to find low-level methylation DNA. There is a report of abnormal methylation detected with MethyLight method in healthy individuals' plasma [21]. Another reason is that we only tested 50 normal individuals which is a relatively small sample size. In addition, we found that the methylation frequency of *GPX3* and *COL14A1* are higher in the pT3 patients compared with pT1–pT2 patients, and the methylation frequency of *EPB41L3* is higher in pN2 patients than in pN0–pN1 patients. These results indicate that not only there are obvious significant difference of aberrant DNA methylation of *EPB41L3*, *GPX3* and *COL14A1* in the plasma between ESCC patients and healthy individuals, but also the methylation frequency might be associated with the advanced tumor stages. Moreover, we also evaluated the methylation status of the 3 genes in plasma for the diagnosis of ESCC using ROC curve analysis. We found that the sensitivities of the methylated genes in plasma DNA range from 31% to 40.5%. A previous study reported that diagnostic information could be increased if methylation of multiple genes in cell-free DNA were analyzed in combination [21]. Indeed, we found that combination analysis of the three genes increased the sensitivity to 64.3%. The sensitivity of the 3 genes for use as early diagnosis tools is not high enough in this study. The combination of more methylated genes may increase the diagnostic sensitivity. These data indicated that combinatorial methylation analysis of these genes in plasma DNA has the potential to be a valuable diagnostic tool of noninvasive testing. The utility of these plasma biomarkers in the clinical setting needs further study in a larger population and with more accurate methods like bisulfite pyrosequencing.
